# Modeling Fractal Structure of City-Size Distributions Using Correlation Functions

**DOI:** 10.1371/journal.pone.0024791

**Published:** 2011-09-20

**Authors:** Yanguang Chen

**Affiliations:** Department of Geography, College of Urban and Environmental Sciences, Peking University, Beijing, China; Universidad Veracruzana, Mexico

## Abstract

Zipf's law is one the most conspicuous empirical facts for cities, however, there is no convincing explanation for the scaling relation between rank and size and its scaling exponent. Using the idea from general fractals and scaling, I propose a dual competition hypothesis of city development to explain the value intervals and the special value, 1, of the power exponent. Zipf's law and Pareto's law can be mathematically transformed into one another, but represent different processes of urban evolution, respectively. Based on the Pareto distribution, a frequency correlation function can be constructed. By scaling analysis and multifractals spectrum, the parameter interval of Pareto exponent is derived as (0.5, 1]; Based on the Zipf distribution, a size correlation function can be built, and it is opposite to the first one. By the second correlation function and multifractals notion, the Pareto exponent interval is derived as [1, 2). Thus the process of urban evolution falls into two effects: one is the *Pareto effect* indicating city number increase (external complexity), and the other the *Zipf effect* indicating city size growth (internal complexity). Because of struggle of the two effects, the scaling exponent varies from 0.5 to 2; but if the two effects reach equilibrium with each other, the scaling exponent approaches 1. A series of mathematical experiments on hierarchical correlation are employed to verify the models and a conclusion can be drawn that if cities in a given region follow Zipf's law, the frequency and size correlations will follow the scaling law. This theory can be generalized to interpret the inverse power-law distributions in various fields of physical and social sciences.

## Introduction

If a region is large enough to encompass a great number of cities, the size distribution of the cities usually follow Zipf's law [1]. Zipf's law for cities is one of the most conspicuous empirical facts in the social sciences [2], [3]. In urban geography, this empirical regularity is also known as the *rank-size rule* [4], [5], [6], [7], [8], [9]. Few social science problems have generated more researches than the urban rank-size distribution of cities, and numerous models have been proposed to account for variations in rank-size regularity. However, many of the plausible explanations stand in direct contradiction to each other [7], [10]. For a long time, there is no convincing explanation for the rank-size rule and the scaling exponent value of city rank-size distribution, despite the frequency with which it has been observed [11]. Today, the rank-size problem seems to be in a dilemma. On the one hand, there are so many theoretical and empirical researches that it seems as if we need no more new models and cases. On the other, the pending problem requires further theoretical study before it will lead us to the underlying rationale of the empirical rule.

In fact, Zipf's law and Pareto distribution are two different sides of the same coin in mathematics [12], [13], [14], but they represent opposite processes of city development in physics. The Pareto distribution is also called Pareto's law since probability distributions are sometimes termed ‘laws’ [15]. Both Pareto's law and Zipf's law can be associated with fractal distribution [16], [17], [18], [19]. Chen and Zhou [20] once proposed a dual multifractals model consisting of multi-Pareto-dimension spectrum and multi-Zipf-dimension spectrum to characterize city size distribution. Generally speaking, a multifactals model is always based on generalized correlation function [21], [22], [23]. Correlation function is one of the very useful tools in urban studies [24], [25]. If we integrate the idea from multifractals, correlation function, and scaling analysis, we can obtain new insight into the rank-size rule of cities and its scaling exponent.

Recent years, a series of interesting studies on or explanations for the rank-size regularity has been published [26], [27], [28], [29], [30], [31], [32]. Especially, the empirical law has been generalized from systems of cities to internal structure of cities as systems, e.g. street hierarchies [33]. These fruits from various fields inspire me to make new researches on city rank-size distribution. This paper will resolve the following problems for the rank-size law. First, I construct two correlation functions based on Pareto's law and Zipf' law, respectively. By scaling analyses, the value intervals of the scaling exponents of the city rank-size distribution are derived. Second, I present a dual competition hypothesis to explain the scaling exponent values, illuminating why the Pareto exponent approaches 1. Third, mathematical experiments and empirical analysis are performed to verify the theoretical models and inferences. In the context, the scaling exponent includes the *Pareto exponent* and the *Zipf exponent*, the former is also called *capacity dimension* or the *zero order correlation dimension*, the latter is also termed *Zipf dimension*, which equals the reciprocal of the Pareto exponent in theory.

## Results

### Discrete correlation functions

Suppose there is a region with *N* cities inside. The size distribution of the *N* cities follows the general form of Zipf's law
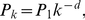
(1) where *P_k_* refers to the population size of the *k*th city, *P*_1_ to the population of the largest city, *k* to the size rank of the *k*th city in the set, and *d*, the scaling exponent, which also called “Zipf dimension” due to its association with fractal dimension of urban hierarchy [17], [23]. If *d* = 1, then equation (1) becomes the pure form of Zipf's law. Zipf's law suggests a Pareto distribution [13], [34]. It is easy to prove that the density function of the Pareto distribution is a special density correlation function (see Text S1)—a kind of hierarchical correlation functions (Figure S1). Let *f*(*x*) represent the number of cities with size over *x*, the discrete correlation function based on the frequency distribution can be defined as
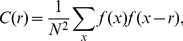
(2)where *x* is the city size scale, *r* denotes a “scale displacement factor”. The measure *x* corresponds to *P_k_* in equation (1), but there is difference: *P_k_* is a concrete value (for the *k*th city), while *x* is a threshold value or scale value (for a class of cities). Equation (2) means that for the cities with size *x*, what is probability of finding the cities with size “*x*-*r*”.

The continuous correlation function can be converted into a discrete correlation function, which can be associated with the gravity model in geography (see Text S2). As stated above, the city size (*x*) is usually measured with urban population (*P*). Now, let *f*(*x*) be fixed as *f*(*x*) = 1 for simplicity. Numbering the cities as *i*, *j* (*i*, *j* = 1, 2, …, *N*), we can reconstruct the above correlation function by means of Zipf's law and yield
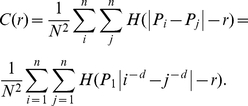
(3)in which *P_i_*, *P_j_* are the size of cities ranked *i* and *j*, and *H*(▪) denotes Heaviside's function, which can be expressed as
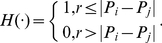
(4)This implies that, without regard to spatial distance, the correlation between two cities is stronger the larger the difference of sizes is (see Table S2). Please notice that, in equations (3) and (4), the ranks *i*, *j* correspond to the variable *k* in equation (1). However, *k* is defined in the 1D space, while *i*, *j* are defined in the 2D space. From equation (2) to equation (3), the frequency correlation is formally replaced by size correlation of cities. The correlation is of scaling invariance if the function follows the power law
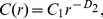
(5) where *r* indicates the “yardmeasure” of city size, *D*_2_ denotes the second order correlation dimension of city size distribution, and *C*_1_ the proportionality coefficient. Actually, we can take *C*_1_ = 1 by normalizing the data. The mathematical experiments and empirical analysis will be performed by using equations (3) to (5) (see Materials and methods).

Generally speaking, correlation function has two mathematical forms (see Figure S2). One is the *correlation density* based on the probability density function, and its mathematical expression is an inverse power law with negative power [35]; the other is the *correlation integral* or *correlation sum* based on the cumulative distribution function, and the mathematical expression is a power law with positive power [36], [37]. The two forms of correlation function can be converted into one another by using differential and integral calculus knowledge [25].

The general correlation model, equation (3), gives a density-density correlation function (the *point-point correlation function*), reflecting the hierarchical correlation between any two cities. Thus, equation (5) presents an inverse power function. If we fix one city, say, *P_j_*, the density-density correlation function will be reduced to a central correlation function (the *one-point correlation function*). In this instance, all cities are correlated with only one city (*P_j_*). Without loss of generality, we may assume *P_j_* = *P*_min_, where *P*_min_ denotes the population of the smallest city in the set. Thus we have a central correlation function
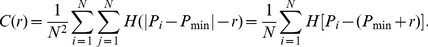
(6)Rescaling the yardstick as *s* = *r*+*P*_min_ yields
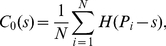
(7)where *s* denotes the rescaled yardmeasure, and the Heaviside function should be rewritten as
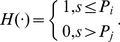
(8)If the central correlation function follows the power law, we deduce Pareto's law such as
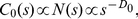
(9)where *D*_0_ refers to the fractal dimension of city-size distribution. This suggests that the Pareto function is a special case of correlation function, and the scaling exponent is in fact the zero order correlation dimension termed “capacity dimension”[25], [37]. By the correlational analysis, Pareto's law and Zipf's law will be integrated into the same framework.

### Continuous correlation functions based on Pareto's law

 The discrete correlation functions are useful in practice, especially in data fitting/analysis and mathematical experiments. However, it is not easy for us to make theoretical transformation and model deduction. In order to derive new parameter relations, we should substitute the continuous form for the discrete form of mathematical models [38], [39]. The function of the Pareto distribution, equations (7) and (9), can be equivalently re-expressed as
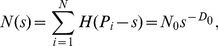
(10)where *N*_0_ denotes the proportionality coefficient. Thus the density function is
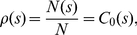
(11)in which, as indicated above, *N* denotes the total number of cities in a region. Based on equation (11), we can construct a continuous density- density correlation function such as
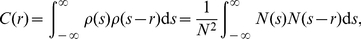
(12)where *r* is the scale factor of city size. This can be termed “Pareto correlation function”, indicating frequency correlation of cities. It is easy to demonstrate that the Pareto correlation function, equation (12), follows the scaling law:
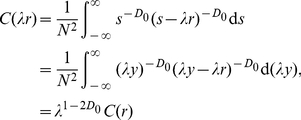
(13)where *λ* refers to a scale factor, and *y* = *s*/*λ* to the replacement of *s*. Variable replacement is a very important technique in scaling analysis of mathematical models. Apparently, the solution to the above functional equation is
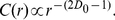
(14)Comparing equation (14) with equation (5) shows the correlation dimension relation as below
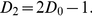
(15)


In theory, a fractal dimension can be treated as a special case/point in the spectrum of generalized correlation dimension, namely, a correlation dimension in a broad sense. In equation (15), *D*_0_ denotes the zero-order correlation dimension (the moment order equals 0), corresponding to the capacity dimension, while *D*_2_ indicates the second order correlation dimension (the moment order is 2), corresponding to the correlation dimension in a narrow sense. The multifractals dimension is a monotonic decreasing quantity with the moment order [17], [21], [22], [40], [41]. Therefore, *D*_0_ is greater than or equal to *D*_2_ for ever, that is, *D*_0_≥*D*_2_. This suggests the first inequation in the form
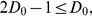
(16)which implies *D*_0_≤1. The numerical relationships between the capacity dimension and the correlation dimension are displayed in Table 1. Obviously, if and only if *D*_0_≤1, we will have *D*_0_≥*D*_2_, and the general fractal dimension spectrum is normal. Otherwise, the multifractals dimension spectrum will fall into disorder. On the other hand, if *D*_0_≤0.5, the correlation dimension *D*_2_≤0, and this is not acceptable in theory. A conclusion can be drawn that the proper capacity dimension of city-size distributions comes between 0.5 and 1, namely, 0.5<*D*_0_≤1.

**Table 1:** The numerical relation between the capacity dimension and the correlation dimension.

Pareto exponent(*D*_0_)	Correlation dimension(*D*_2_)	Zipf exponent(*d*_0_)	Zipf's correlation exponent(*d*_2_)
**0.5**	**0**	2	3
**0.6**	**0.2**	1.667	2.333
**0.7**	**0.4**	1.429	1.857
**0.8**	**0.6**	1.250	1.500
**0.9**	**0.8**	1.111	1.222
**1**	**1**	**1**	**1**
1.1	1.2	**0.909**	**0.818**
1.2	1.4	**0.833**	**0.667**
1.3	1.6	**0.769**	**0.538**
1.4	1.8	**0.714**	**0.429**
1.5	2	**0.667**	**0.333**
1.6	2.2	**0.625**	**0.250**
1.7	2.4	**0.588**	**0.176**
1.8	2.6	**0.556**	**0.111**
1.9	2.8	**0.526**	**0.053**
2	3	**0.500**	**0**

**Note**: The bold denotes the rational intervals of the scaling exponent values.

### Continuous correlation functions Based Zipf's law

The above frequency correlation is based on Pareto's density distribution function. In fact, from another perspective, we can also construct a hierarchical correlation function based Zipf's law. Generalizing the discrete rank variable (*k*) in equation (1) to a continuous metric variable, we may define a density function *p*(*k*) = *P*(*k*)/*P*, where *P*(*k*) refers to the size of the *k*th city, and *P* to the total urban population. Thus, a correlation function can be made as
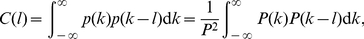
(17)in which *l* represents a scale factor of city rank. This can be termed “Zipf correlation function” indicative of size correlation of cities. For simplicity, we don't change the symbol *k* for the conversion from discrete distribution to continuous process. By analogy with equation (13), we can prove that the Zipf correlation function, equation (17), satisfies the following scaling relation
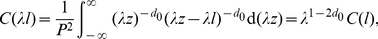
(18)where *z* = *k*/*λ* is the substitute of *k*, and *d*_0_ is used to replace *d* to denote the zero order Zipf dimension. The solution to equation (18) is a power function as
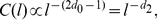
(19)where the scaling exponent *d*_2_ represents the second order Zipf dimension [20], which can be expressed as
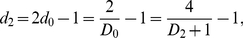
(20)in which *d*_0_ = 1/*D*_0_. It is easy to prove that the Zipf exponent is the reciprocal of the capacity dimension of city size distribution [23].

The Zipf dimension spectrum is also a monotonic decreasing quantity with the moment order [20]. Thus we have *d*_0_≥*d*_2_. So, equation (20) suggests the second inequation such as
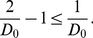
(21)That is, *D*_0_≥1. The numerical relationships between the zero order Zipf dimension and the second order Zipf dimension are listed in Table 1. Apparently, when and only when *D*_0_≥1 or *d*_0_≤1, we have *d*_0_≥*d*_2_, and the Zipf dimension spectrum is normal. Or else, the Zipf dimension spectrum will fall into confusion. On the other hand, if *d*_0_≤0.5 or *D*_0_≥2, the second order Zipf dimension *d*_2_≤0, and this is meaningless in theory. The conclusion can be reached that the proper Zipf dimension of city-size distributions also falls between 0.5 and 1, namely, 0.5<*d*_0_≤1; accordingly, we have 1≤*D*_0_<2. Combining the two inequalities, relations (16) and (21), yields
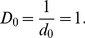
(22)This suggests that, in order to satisfy the rationality of both the Pareto dimension spectrum and the Zipf dimension spectrum at the same time, the scaling exponent of the rank-size distribution must be equal to 1 in theory and close to 1 in practice.

### A dual competition process of city development

In mathematical form, Zipf's law is equivalent to Pareto's law. However, the parameter-value interval (1≤*D*_0_<2) derived from the Zipf's-law-based correlation model differs from that (0.5<*D*_0_≤1) derived from the Pareto's-law-based correlation model. This suggests that Zipf's law is actually related to but different from Pareto's law in physical meaning. This is an interesting finding for our understanding urban evolution. In reality, city development in a region consists of two major, apparently contradictory, but essentially compatible, processes. One is that cities try to become more and more in number, the other is that each city tries to become larger and larger in size. The former is a process of city number increase indicating *external complexity* at the macro level, whereas the latter is a process of city size growth indicating *internal complexity* at the micro level. The concepts of external and internal complexity came from biology [42]. The former can be termed *Pareto effect*, while the latter, termed *Zipf effect*. The two processes of urban evolution always come into unity of opposites. In theory, the Zipf distribution can be transformed into a self-similar hierarchy, and the competitive relations between city number and city size follows the inverse power law such as
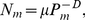
(23)where *m* is the class/level order in an urban hierarchy (*m* = 1, 2, 3,…), *N_m_* refers to the number of cities in the *m*th level, *P_m_* to the average size of the *N_m_* cities, *μ* to the proportionality coefficient, and *D*, to the fractal dimension of the self-similar hierarchy. Equation (23) can be decomposed into two processes: city number (*N_m_*) increase and city size (*P_m_*) growth [38].

In fact, many evidences support the judgment that city development bears a dual nature, which seems to result from the laws of the unity of opposites. The first is the previous empirical studies, which showed a contradictive process of urban evolution: both city number increase and city size growth are subject to the total urban population [9], [43]. The second is the entropy maximization of urban evolution, which falls into two competitive processes: frequency distribution entropy maximizing and size distribution entropy maximizing [44]. The former corresponds to the Pareto effect while the latter to the Zipf effect. The third is the simulation experiments by means of geographical cellular automata (CA), which revealed that urban evolution seems to express a struggle between two opposite processes [45], [46]. The correlation functions presented above can be employed to reflect the “contradiction” or the “unity of opposites” in urbanization.

Now, a new hypothesis on the dual competition of city development is proposed as follows. If the Pareto effect plays the leading role in evolution of urban systems, the fractal dimension *D*_0_ comes between 0.5 and 1; accordingly, the Zipf dimension ranges from 1 to 2. In contrast, if the Zipf effect plays a dominant part in city development, the fractal dimension *D*_0_ comes between 1 and 2, and consequently, the Zipf dimension varies from 0.5 to 1. If the two effects reach equilibrium with each other, the scaling exponents *D*_0_ or *d*_0_ approaches 1, that is, *D*_0_ = 1/*d*_0_→1. On the other hand, if *D*_0_≤1 or *d*_0_≥1, the Pareto dimension spectrum is normal, but the Zipf dimension spectrum is abnormal; if *D*_0_≥1 or *d*_0_≤1, the Zipf dimension spectrum is rational, but the Pareto dimension spectrum is illogical. The composition of forces of the two effects always leads the scaling exponent to the unit: *D*_0_ = 1/*d*_0_ = 1. What is more, the positions of the Pareto effect and the Zipf effect can be in exchange with each other. As soon as the scaling exponent go from one extreme to the other (say, from to *D*_0_>1 to *D*_0_<1), one effect will change to another effect.

## Materials and Methods

### Mathematical experiments

One of the key points in this paper is such a conjecture that if the size distribution of cities follows Zipf's law, the hierarchical correlation function will follow the scaling law. This has been theoretically proved by scaling analysis in Results, based on continuous variables of city rank and size. Now, let's make mathematical experiments based on discrete variables to verify the abovementioned inference. As an example, let *N* = 500, that is, consider 500 cities in a region. Suppose that all these cities meet the rank-size distribution defined by equation (1). Thus the city sizes can be abstracted as *p*-sequence such as {1, 1/2*^p^*, 1/3*^p^*, …, 1/500*^p^*}, where *p* denotes a subset of *d*. The “yardstick” *r* ranges from 0 to 1 and the step length of yardstick change is taken as Δ*r* = 1/32, that is, *r* = (0), 1/32, 2/32, …, 31/32, (1).

In empirical analyses, for simplicity and lucidity, equation (3) can be equivalently replaced by
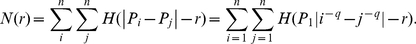
(24)Correspondingly, equation (5) can be rewritten as
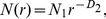
(25)where *N*_1_ = *C*_1_ *N* denotes a proportionality constant. This is to say, if we substitute correlation number *N*(*r*) for correlation density *C*(*r*), the scaling exponent will not change [25]. We can employ some kind of computer software such as Matlab to carry out the mathematical experiments.

**Figure 1:**
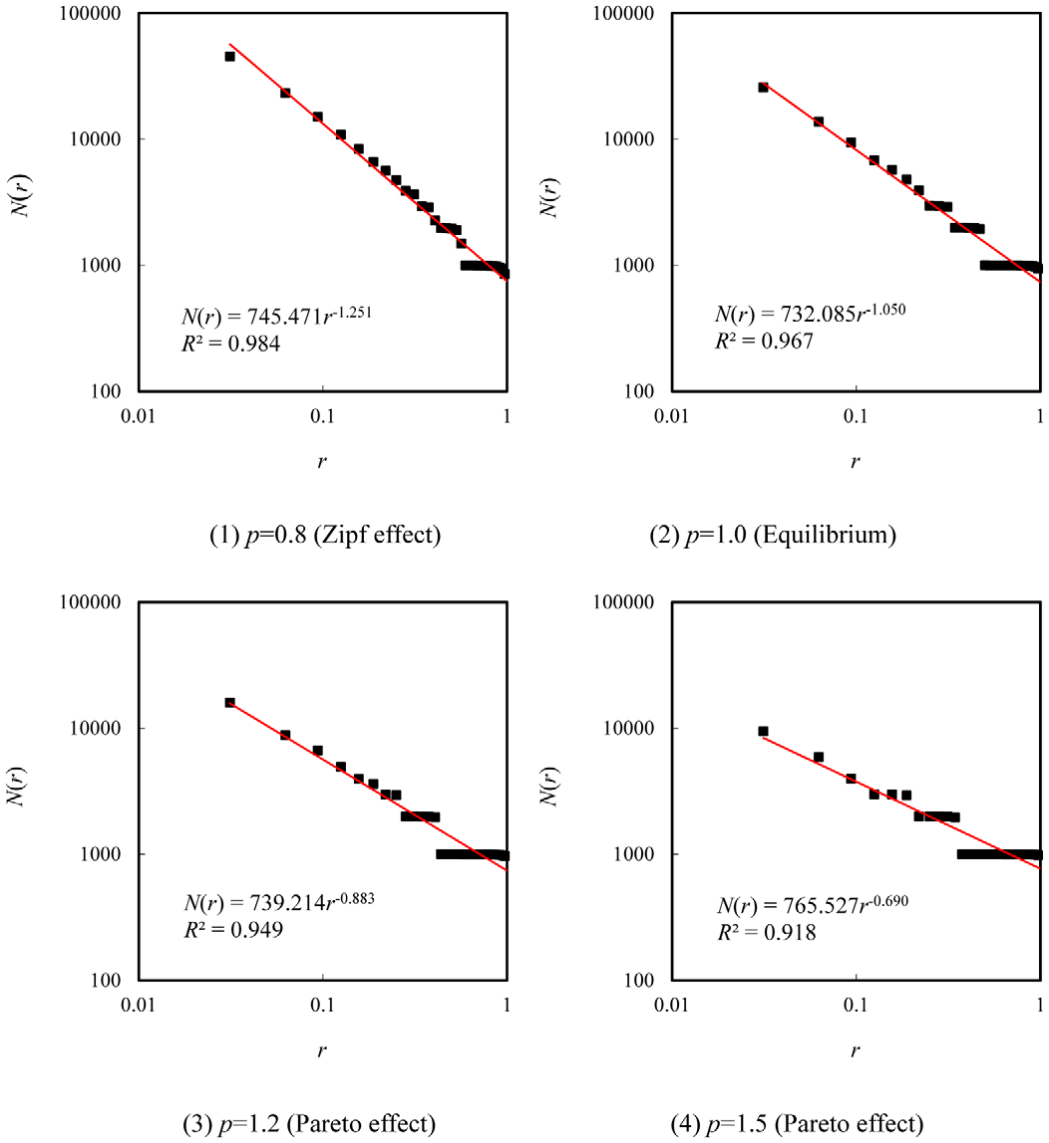
Four typical patterns of size correlation of cities measured by yardstick and correlation number.

The experiment results shows that the relations between yardstick *r* and correlation number *N*(*r*) follow the scaling law (Figure 1). The scaling exponents give the second order correlation dimension *D*_2_ values. The zero order correlation dimension, i.e., capacity dimension, can be estimated with equation (15), that is *D*_0_ = (*D*_2_+1)/2. Changing *p* value of the *p*-sequence bears an analogy to change the *d* value in equation (1). The expected capacity dimension is *D*_0_^*^ = 1/*d* = 1/*p*. There are always errors between the theoretical values derived from the correlation models with continuous variables and the corresponding computational results based on discrete variables from observations or experiments [38]. The squared error between computational capacity dimension and expected capacity dimension can be defined as *e* ^2^ = (*D*_0_−*D*_0_^*^)^2^. Parts of these results from the least square computation are listed in Table 2 for reference.

**Table 2:** Partial results of mathematical experiments for hierarchical correlation analysis of city rank-size distributions.

*p*	0.6	0.7	0.8	0.9	1.0	1.1	1.2	1.3	1.4	1.5	2.0
*D*_2_	1.5247	1.3693	1.2510	1.1466	1.0495	0.9618	0.8826	0.8158	0.7473	0.6903	0.4544
*R* ^2^	0.9727	0.9852	0.9836	0.9777	0.9668	0.9589	0.9489	0.9362	0.9335	0.9184	0.8199
*D*_0_	1.2624	1.1847	1.1255	1.0733	1.0248	0.9809	0.9413	0.9079	0.8737	0.8452	0.7272
*D*_0_^*^	1.6667	1.4286	1.2500	1.1111	1.0000	0.9091	0.8333	0.7692	0.7143	0.6667	0.5000
*e* ^2^	0.1635	0.0595	0.0155	0.0014	0.0006	0.0052	0.0117	0.0192	0.0254	0.0319	0.0516

**Note**: *R* ^2^ denotes the correlation coefficient square, i.e., the goodness of fit.

From the process and results of the mathematical experiments, we can come to the following judgments. First, if *p*≤0.5, the size correlation experiments cannot be implemented, or there is no hierarchical correlation. This suggests *d*_0_>0.5, and thus *D*_0_ = 1/*d*<2. Second, only if 1≤*d*_0_<2, that is, 1≥*D*_0_>0.5, we will have *D*_0_≥*D*_2_>0. Otherwise, the multifractals dimension spectrum or the dimension relations will fall into disorder. Third, when *d*_0_≈1, and thus *D*_0_≈*D*_2_≈1, the computation results is most consistent with the theoretical derivation. Actually, when *d*_0_→1, or *D*_0_→1, the squared error of computational capacity dimension approaches the minimum. If *d*_0_<<1, the scaling relation tends to be broken down; if *d*_0_>>1, the deviation extent of scaling relation become very large (Figure 1). The conclusions can be drawn as below. First, the value range of the scaling exponent is 0.5<*d*_0_<2, or 2>*D*_0_>0.5. Second, the standard rank-size distribution described by the *p*-sequence is a monofractal distribution rather than a multifractal distribution, and thus the expected fractal dimension is *D*_0_ = *D*_2_ = 1, and the corresponding Zipf dimension is *d*_0_ = *d*_2_ = 1. In short, the mathematical experiments support the results from the correlational analyses fully.

### Empirical evidences

**Figure 2:**
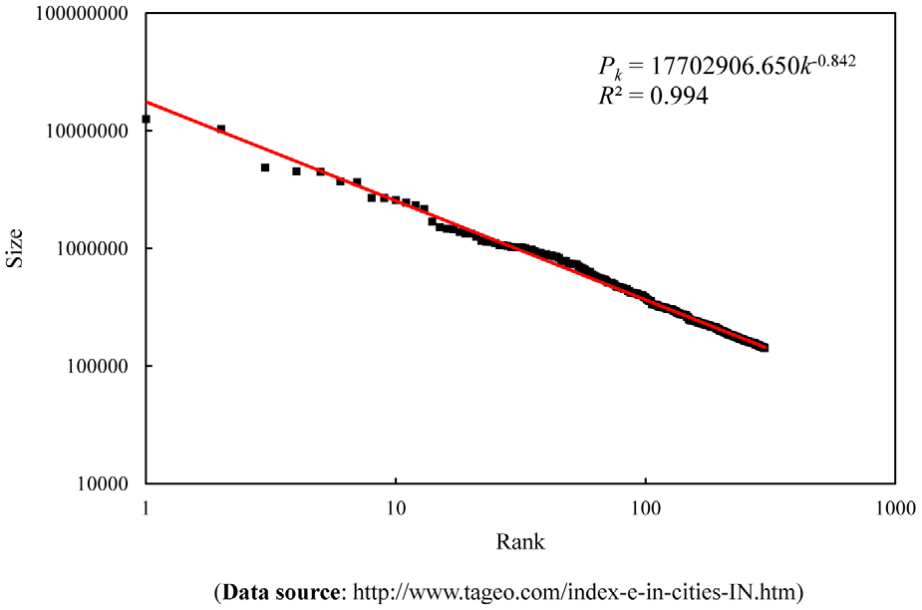
The rank-size pattern of the first 513 US cities in 2000 (The trend line is given by the least square computation).

**Figure 3:**
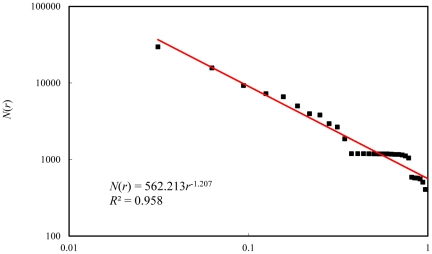
The hierarchical correlation patterns of the U.S. cities based on UA population in 2000.

As an empirical case, the cities of the United States of America (USA) are employed to make a hierarchical correlation analysis. The population in urbanized area (UA) is always used to measure the city sizes of America. The 513 largest cities with UA population over 40,000 in the year of 2000 are available from the U.S. Census Bureau's website (see: http://en.wikipedia.org/wiki/). These cities comply with Zipf's law approximately and thus take on a rank-size distribution (Figure 2). On the whole, the correlation function follows the scaling law (Figure 3). Using the least square computation, we can estimate the capacity dimension *D*_0_ and the correlation dimension *D*_2_. By means of equation (1), the capacity dimension is estimated as *D*_0_ = 1/*d*≈0.878, and the goodness of fit is about *R* ^2^ = 0.990; By means of equation (24) and (25), the correlation dimension is estimated as *D*_2_≈1.296, and the goodness of fit is around *R* ^2^ = 0.977. This implies *D*_0_<*D*_2_, and the result is abnormal. However, if we replace the least square method with the nonlinear fit method, the results are *D*_0_≈1.225, *D*_2_≈1.012, respectively. This time, *D*_0_>*D*_2_, and this seems to be normal. As is often the case, different algorithms yield different results and then lead to different conclusions.

The least square method benefits the medium-sized cities and small cities (Figure 2), while the nonlinear fit method favors large cities. In fact, the rank-size distribution can be transformed into a self-similar hierarchy, and then we can estimate the fractal dimension of the city-size distribution with the generalized 2*^n^* rule [17], [38]. By the 2*^n^* principle, the capacity dimension is estimated as *D*_0_≈0.992 (*d*_0_≈1.008), and *R* ^2^ = 0.990. In this instance, the correlation dimension is expected to approach 1, that is, *D*_2_≈*D*_0_≈1 (Table 3). The self-similar hierarchy can filter the random disturbance of various noises so that the result is more stable and dependable. It can be seen that the size sequence of cities in the real world is more complicated than the *p*-sequence in the mathematical world.

**Table 3:** Different approaches to estimating the fractal dimension values of the U.S. city-size distribution (2000).

Approach	Algorithm	Relation	Result	Judgment
Rank-size distribution	Least square	Log-linear	*D*_0_≈0.878, *D*_2_≈1.296	Abnormal
	Nonlinear fit	Nonlinear	*D*_0_≈1.225, *D*_2_≈1.012	Normal
Self-similar hierarchy	Least square	Log-linear	*D*_0_≈0.992, *D*_2_≈0.984	Normal

Applying the hierarchical correlational analysis to the U.S. cities gives us an insight into the city-size distributions. For the 513 top U.S. cities, the data points actually follow along two trend lines with different slopes. The large cities in the minority (about 32 cities) share one trend line (the slope is *d*_0_≈0.763, thus *D*_0_≈1.311), while the medium-sized and small cities (about 481 cities) in the majority share another one (the slope is *d*_0_≈1.235, thus *D*_0_≈0.810). This suggests that the large U.S. cities took on Zipf's effect, but the medium and small cities presented the Pareto effect. The large cities tried to become larger, while the medium and small cities tried to become more than ever. However, where statistical average is concerned, the two effects seem to be balanced. In theory, in order to reconcile the two effects of city development, the scaling exponents (*d*_0_, *D*_0_) approach 1 in the process of urbanization. But for the real cities, the competition of the two effects often breaks the scaling relation so that one tread line divides into two on log-log plots. The cause of the scaling breaking may be as follows. People attach more importance to and invest heavily in large cities, but the geographical space and natural environment cannot support the grand-scale population of a city. On the other, people pay less attention to and invest lightly to smaller cities, which are usually undergrown. The result is that both the largest cities and smallest cities fail to reach the sizes predicted by the pure form of Zipf's law (*d* = 1). In fact, the correlational model can also be applied to the cities in the developing countries such as China and India (see Text S3). For example, the Indian cities follow Zipf's law (Figure S3), and thus the hierarchical correlation follows the power law (Figure S4). By empirical analyses, we can draw a comparison between the U.S. cities and the systems of cities of China and India (see Table S2).

## Discussion

The hierarchical correlation models are built for understanding the city rank-size distribution and its scaling exponent. Neumann [47, page 492], one of the greatest scientists in the 20^th^ century, once pointed out: “The sciences do not try to explain, they hardly even try to interpret, they mainly make models.” Zipf's law is one of the scaling laws in nature and society [6], [38], and scaling laws often typically reflect or even reveal the general principles underlying the structure of a physical problem [48]. The Zipf dimension as well as the Pareto exponent can be treated as a kind of scaling exponent. In order to bring to light the fundament of urban systems, we must estimate the Pareto exponent *D*_0_ or Zipf's dimension *d*_0_. However, a number of factors affect the parameter estimation. Among these factors, the main are algorithms and city definition. The algorithms in common use include the least square method, the maximum likelihood method, and the nonlinear fit method. The maximum likelihood estimation requires the data meet the normal distribution. Matlab directly provides two algorithms: the least square and nonlinear fit. The former is based on logarithmic scale (geometric scale), while the latter based on conventional scale (arithmetic scale). If we use the nonlinear fit method, the large cities in the minority will affect the parameter's estimated value; if we use the least square method, the medium and small cities in the majority will influence the result. The essence of algorithmic effect rests with structure of city-size distributions. If the data points distribute along a single straight line on the log-log plot, the results of parameter estimation from different algorithms should be very close to one another. The U.S. cities seem to form two straight lines rather than one trend line on the logarithmic plot (Figure 2). If we transform the rank-size distribution into a self-similar hierarchy, the problems stemming from algorithms can be well resolved in empirical analyses.

The definition of cities is an important factor impacting the estimation of scaling exponents. In China, there has been no normal or standard definition for cities so far [49]. In USA, there are three basic concepts used to define urban areas and populations, namely, city proper (CP), urban agglomeration or urbanized area (UA), and metropolitan area (MA) [50]. The most appropriate one may be UA because it leads to the compatible relations between Zipf's law and the allometric growth law of cities. However, I suggest that the concept of “natural cities” defined by Dr. Jiang and his coworkers should be adopted for urban scaling analysis, because this definition of cities is the most objective one among varied city definitions in use [51], [52], [53]. The objectivity of city definition is one of the preconditions guaranteeing the validity of fractal dimension analysis for city rank-size distributions in practice.

If there is no problem in algorithms and urban definition, the scaling exponents, including the Pareto exponent (capacity dimension) and the Zipf dimension, can be employed to make an analysis of city development. The Pareto exponent coming between 0.5 and 1 suggests that the Pareto effect gain an advantage over the Zipf effect, and the cities try to become more in number (external complication); The Pareto exponent falling between 1 and 2 implies that the Zipf effect get an advantage of the Pareto effect, and each city tries to become larger (internal complication) (Table 4). The competition of the two effects leads to two possible results: one is state of equilibrium with scaling exponent equal to 1, and the other is scaling break and data points distribute along two straight lines with different slopes instead of an exclusive line in log-log plots. The scaling pattern of the 513 U.S. cities is actually broken into two lines to some extent, but as a whole, it can be approximately treated as a straight line (Figure 2).

**Table 4:** Two effects and two hierarchical correlation processes in evolution of urban systems.

Effect	Correlation function	Meaning	Behavior	Complexity	Multifractals spectrum	Parameter interval
Pareto effect	Pareto correlation	Frequency correlation	City number increase	External complexity	General fractal dimension	0.5<*D*_0_≤1,1≤*d*_0_<2
Zipf effect	Zipf correlation	Size correlation	City size growth	Internal complexity	Zipf dimension	0.5<*d*_0_≤1,1≤*D*_0_<2

**Note**: The general fractal dimension is also called the Pareto-dimension spectrum in the context.

Urban systems differ from the classical physical systems. Only behavioral studies are necessary for physical systems. However, for urban systems, we need not only *behavioral analysis* but also *normative analysis*. The behavioral theories are on the real patterns of cities, while the normative theories are about the ideal structure of cities and systems of cities, which provide us a rational value judgment and problem diagnosis for city development. The hierarchical correlational analysis of cities is mainly a normative theory, which tells us what are the reasonable intervals of the scaling exponents of city-size distributions, and what is the distance from the real state to the ideal state of urban evolution. It is impossible to clarify many questions at a time. Some problems remain to be resolved in future. The sum of this paper is as follows. (1) For the first time, Zipf's law is distinguished from Pareto's law in physics, and the proper scales of the scaling exponents are revealed. Zipf's law is mathematically equivalent to Pareto's law, but they represent a dual process in urban evolution. Based on Pareto's law, we can construct a frequency correlation function, from which follows a rational value interval of the Pareto exponent as (0.5, 1]; based on Zipf's law, we can construct a size correlation function, from which follows another rational interval of the Pareto exponent as [1, 2). The intersection of the two intervals is *D*_0_ = 1/*d*_0_ = 1. (2) The dynamical mechanism of city development or urban evolution is reduced to two effects. One is the Pareto effect associated with frequency correlation, the other is the Zipf effect associated with size correlation. The two effects are of unity of opposites. If the Pareto effect plays the leading role in urban evolution, cities try to become more and more in number; if the Zipf effect plays a dominant part in city development, each city tries to become larger and larger in size. (3) The scaling exponent of Zipf's law or Pareto's law equal to 1 indicates the optimum structure of city-size distribution. It is hard for urban evolution to satisfy both sides of the two effects. If the Pareto effect win the advantage over the Zipf effect, the multifractals dimension (multi-Pareto-dimension) spectrum will be rational, but the multi-Zipf-dimension spectrum will be illogical; if the Zipf effect has advantage of the Pareto effect, the multi-Zipf-dimension spectrum will be normal, but the multifractal spectrum will be abnormal. The result of competition of the two effects is either scaling break or the scaling exponent close to 1.

## Supporting Information

Figure S1 Temporal, spatial, and hierarchical correlation functions of cities.
(TIF)

Figure S2 Two forms of a correlation function and the relation between them.
(TIF)

Figure S3 The rank-size pattern of the first 300 Indian cities in 2000 (The trend line is given by the least square computation).
(TIF)

Figure S4 The hierarchical correlation patterns of Indian cities in 2000.
(TIF)

Table S1 The size difference and size product of a ten-level hierarchy of cities (Examples for hierarchical lags 1 and 2).
(DOCX)

Table S2 Comparison between the US cities, India's cities and China's cities in 2000.
(DOCX)

Text S1 **There types of correlation functions of cities.**
(DOCX)

Text S2 **The gravity model, spatial correlation, and hierarchical correlation.**
(DOCX)

Text S3 **An application of hierarchical correlation model to Indian cities.**
(DOCX)
