# Supplementary figures and images for: Modeling Fractal Structure of City-Size Distributions Using Correlation Functions
Source: PLoS One. 2011 Sep 20;6(9):e24791. doi: 10.1371/journal.pone.0024791 (PMC3176775; doi:10.1371/journal.pone.0024791)

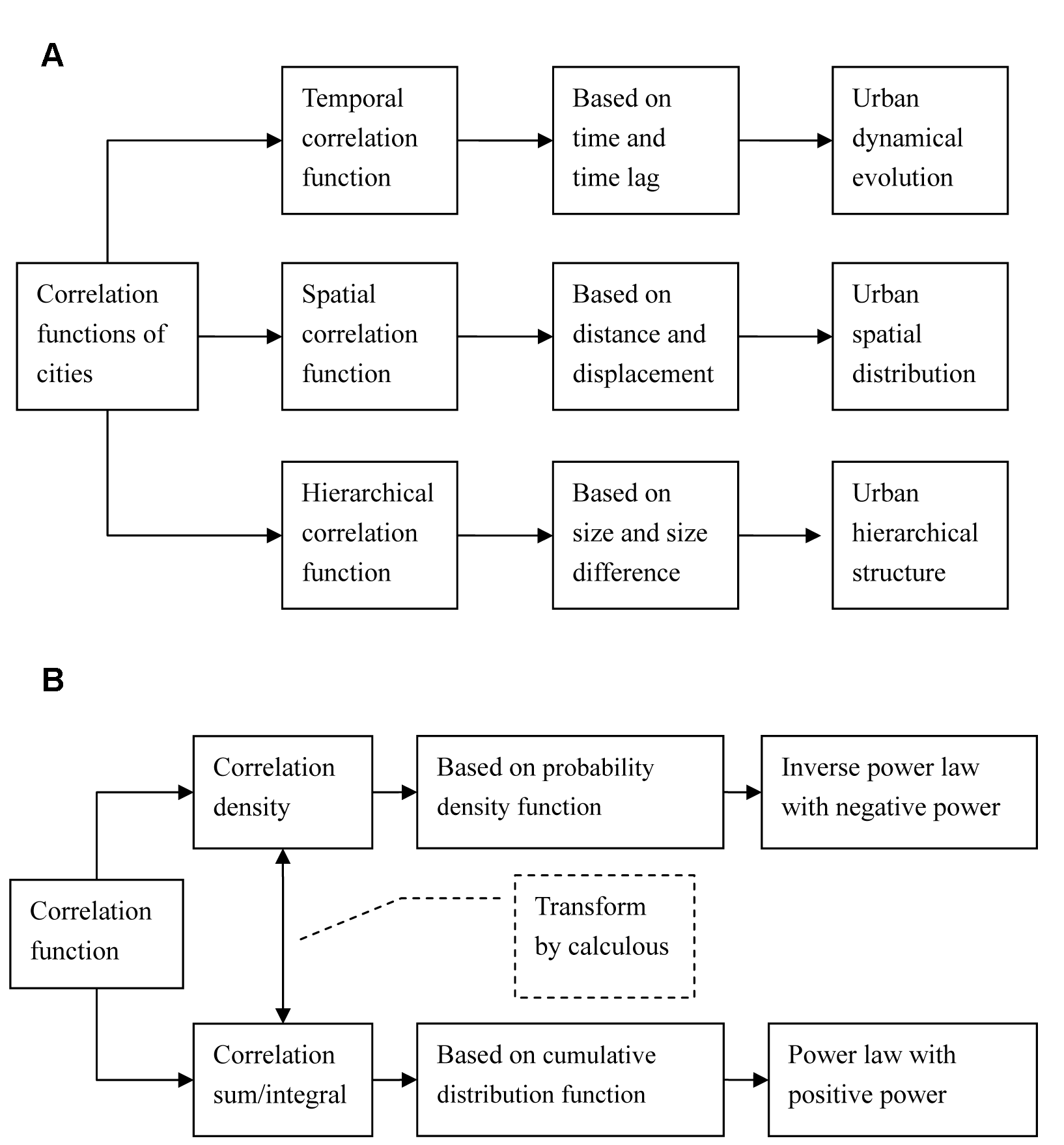

Supplement: Figure S1 — Temporal, spatial, and hierarchical correlation functions of cities. (TIF) [file pone.0024791.s001.tif]

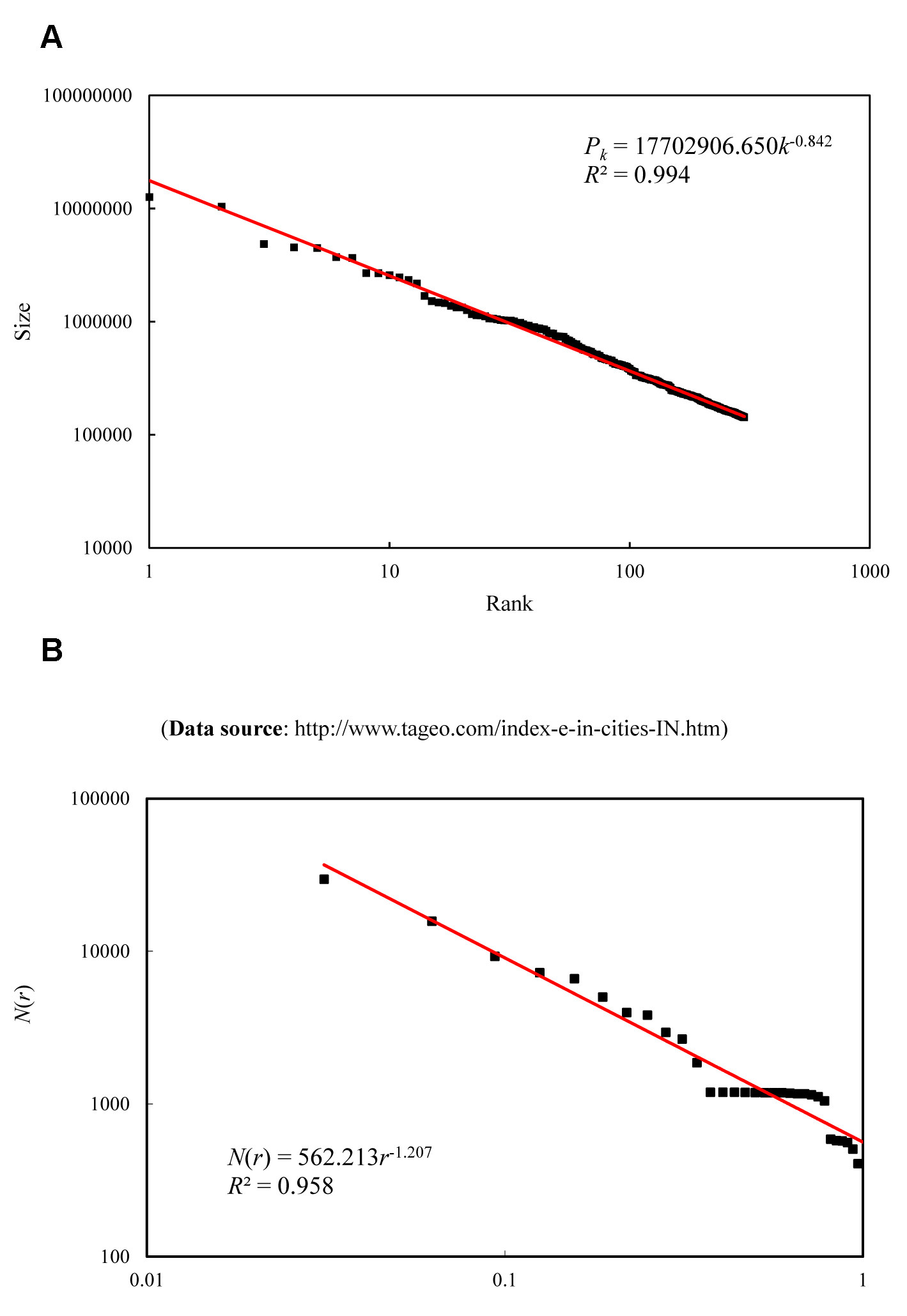

Supplement: Figure S2 — Two forms of a correlation function and the relation between them. (TIF) [file pone.0024791.s002.tif]

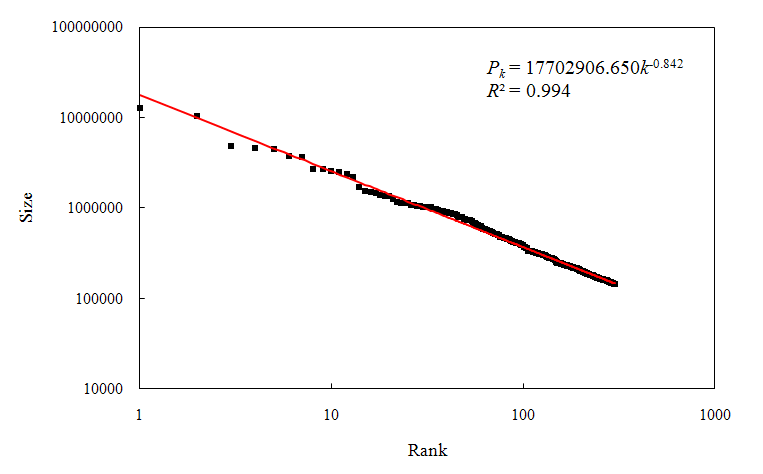

Supplement: Figure S3 — The rank-size pattern of the first 300 Indian cities in 2000 (The trend line is given by the least square computation). (TIF) [file pone.0024791.s003.tif]

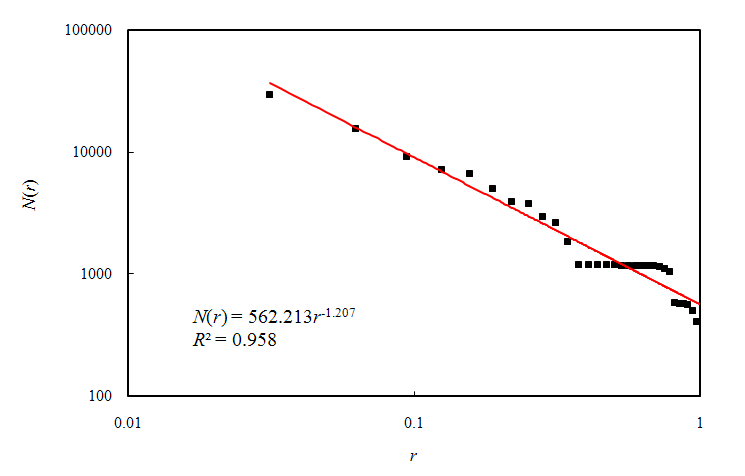

Supplement: Figure S4 — The hierarchical correlation patterns of Indian cities in 2000. (TIF) [file pone.0024791.s004.tif]
