# Supplementary material for: Modeling Fractal Structure of City-Size Distributions Using Correlation Functions
Source: PLoS One. 2011 Sep 20;6(9):e24791. doi: 10.1371/journal.pone.0024791 (PMC3176775; doi:10.1371/journal.pone.0024791)
Supplement: Table S1 — The size difference and size product of a ten-level hierarchy of cities (Examples for hierarchical lags 1 and 2). (DOCX) [file pone.0024791.s005.docx]

**Table S1** The size difference and size product of a ten-level hierarchy of cities (Examples for hierarchical lags 1 and 2)

| *m* | *P_m_* | lag=1 | | lag=2 | |
| --- | --- | --- | --- | --- | --- |
|  |  | *P_m_*-*P_m_*_+1_ | *P_m_***P_m_*_+1_ | *P_m_*-*P_m_*_+2_ | *P_m_***P_m_*_+2_ |
| 1 | 1 |  |  |  |  |
| 2 | 0.5 | 0.5 | 0.5 |  |  |
| 3 | 0.25 | 0.25 | 0.125 | 0.75 | 0.25 |
| 4 | 0.125 | 0.125 | 0.03125 | 0.375 | 0.0625 |
| 5 | 0.0625 | 0.0625 | 0.0078125 | 0.1875 | 0.015625 |
| 6 | 0.03125 | 0.03125 | 0.001953125 | 0.09375 | 0.00390625 |
| 7 | 0.015625 | 0.015625 | 0.00048828125 | 0.046875 | 0.0009765625 |
| 8 | 0.0078125 | 0.0078125 | 0.0001220703125 | 0.0234375 | 0.000244140625 |
| 9 | 0.00390625 | 0.00390625 | 0.000030517578125 | 0.01171875 | 0.00006103515625 |
| 10 | 0.001953125 | 0.001953125 | 0.00000762939453125 | 0.005859375 | 0.0000152587890625 |

**Note**: In the table, *m* is the order number of city class/level (*m*=1, 2, 3,…,), *P_m_* refers to the average size of order *m*, *P_m_*-*P_m_*_+_*_h_* to the size difference, and *P_m_***P_m_*_+_*_h_* to the size product. Here *h*=1, 2, 3,… denotes hierarchical lag.
