# Supplementary material for: Modeling Fractal Structure of City-Size Distributions Using Correlation Functions
Source: PLoS One. 2011 Sep 20;6(9):e24791. doi: 10.1371/journal.pone.0024791 (PMC3176775; doi:10.1371/journal.pone.0024791)
Supplement: Table S2 — Comparison between the US cities, India's cities and China's cities in 2000. (DOCX) [file pone.0024791.s006.docx]

**Table S2** Comparison between the US cities, India’s cities and China’s cities in 2000

| Cities | Zipf dimension | Principal effect | Scaling relation | Urbanization |
| --- | --- | --- | --- | --- |
| The US cities | *d*_0_≈1/*D*_0_≈1 | Two effects are approximately equal | Break to some extent | Terminal stage |
| India’s cities | *d*_0_≈1/*D*_0_<1 | Zipf effect plays the leading role | No significant break | Acceleration stage |
| China’s cities | *d*_0_≈1/*D*_0_<1 | Zipf effect plays the leading role | Break to a degree | Acceleration stage |
