# Supplementary material for: Modeling Fractal Structure of City-Size Distributions Using Correlation Functions
Source: PLoS One. 2011 Sep 20;6(9):e24791. doi: 10.1371/journal.pone.0024791 (PMC3176775; doi:10.1371/journal.pone.0024791)
Supplement: Text S1 — There types of correlation functions of cities. (DOCX) [file pone.0024791.s007.docx]

## Text S1 There types of correlation functions of cities

The correlation functions of cities fall into three classes—*temporal correlation function* (TCF), *spatial correlation function* (SCF), and *hierarchical correlation function* (HCF) (see Figure S1). The TCF is simple and easy to understand. The SCF and HCF of cities are more interesting but it is more difficult to study them. In this paper, I study HCF based on the rank-size distribution of cities. Each correlation function, TCF, SCF, or HCF, has two mathematical forms--the *correlation density* based on the inverse power law with a negative power and the *correlation integral*/*sum* based on the power law with a positive power. The two forms of correlation function are equivalent to one another in mathematics (Figure S2). For the spatial correlation of cities, the fractal dimension can be directly estimated by correlation integral/sum. For the hierarchical correlation, however, we have three kinds of fractal dimension--*network dimension*, *population distribution dimension*, and *rank-size distribution dimension*. If we estimate the network dimension or population distribution dimension, we should adopt correlation integral/sum function; if we calculate the rank-size distribution dimension, we should use correlation density function.
