# Supplementary material for: Modeling Fractal Structure of City-Size Distributions Using Correlation Functions
Source: PLoS One. 2011 Sep 20;6(9):e24791. doi: 10.1371/journal.pone.0024791 (PMC3176775; doi:10.1371/journal.pone.0024791)
Supplement: Text S2 — The gravity model, spatial correlation, and hierarchical correlation. (DOCX) [file pone.0024791.s008.docx]

## Text S2 The gravity model, spatial correlation, and hierarchical correlation

The HCF is contrary to the SCF where the effect is concerned. For the spatial distribution of cities, the correlation is stronger the smaller the distance between two cities is. However, for the hierarchy of cities, the correlation is stronger the larger the difference of city sizes is. The judgment is consistent with the gravity model in social science, which can be expressed as

$I_{ij}=G\frac{P_{i}P_{j}}{L_{ij}^{b}}$, (S1)

where *I* refers to the interaction strength, *P* to the population size of each city, *L* to the spatial distance, and *G* and *b* to constants. This implies that any two cities act on one another with an attraction that is proportional to the product of their sizes and inversely proportional to the *b*th power of the distance between them. The correlation strength is proportional to the product of size measures, *P_i_* and *P_j_*. On the other hand, the size product, *P_i_***P_j_*, is proportional to size difference, *P_i_*-*P_j_*. Let’s see a simple example (Table S1). Suppose that there exists a system of cities following Zipf’s law. The cities can be grouped in 10 classes according to the 2*^n^* rule of Davis (1978). Obviously, for given hierarchical lag, the larger the size difference is, the larger the size product is. In light of the gravity model, the larger the size product is, the stronger the interaction/correlation is.

The gravity model can be transformed into a correlation function on certain condition. This suggests that the interaction is related to the correlation of cities. The simplest case is based on the 2*^n^* principle (Davis, 1978). Suppose a set of cities in a region follows Zipf’s law. We can group these cities in different classes, which form a self-similar hierarchy (Chen, 2010). For different levels of the hierarchy of cities, let *P_i_*=*f*(*m*), and *P_j_*=*f*(*m*+*h*), where *m* refers to order of level, and *h* to the hierarchical lag. In theory, the discrete variables can be treated as continuous variable, thus equation (S1) can be rewritten as

$I_{ij}(h)=G\frac{f_{i}(m)f_{j}(m+h)}{L_{ij}^{b}}$, (S2)

The integral of *I_ij_* with respect to *m* is

$F(h)=\int I_{ij}(h)\text{d}m=\frac{G}{L_{ij}^{b}}\int f_{i}(m)f_{j}(m+h)\text{d}m=\frac{G}{L_{ij}^{b}}C(h)$, (S3)

where

$C(h)=\int f_{i}(m)f_{j}(m+h)\text{d}m$ (S4)

is just a correlation function. By the self-similar hierarchy, a cross correlation function (CCF) can be converted into autocorrelation function (ACF), and a complicated problem can be simplified in mathematics. More relationships between the gravity model and correlation functions can be revealed by theoretical derivation and analysis.
