# Supplementary material for: Modeling Fractal Structure of City-Size Distributions Using Correlation Functions
Source: PLoS One. 2011 Sep 20;6(9):e24791. doi: 10.1371/journal.pone.0024791 (PMC3176775; doi:10.1371/journal.pone.0024791)
Supplement: Text S3 — An application of hierarchical correlation model to Indian cities. (DOCX) [file pone.0024791.s009.docx]

## Text S3 An application of hierarchical correlation model to Indian cities

The hierarchical correlational analysis can be applied to other systems of cites such as India’s cities and China’s cities. Let’s see Indian system of cities. The statistical dataset of the top 300 cities of India in 2000 are available from Indian website (http://www.tageo.com/). The cities follow Zipf’s law (Figure S3), and a least square computation yields the following model

$P_{k}=1.7702906.650k^{-0.842}$.

The goodness of fit is about *R*^2^=0.994, and the Zipf dimension of city-size distribution is estimated as *d*_0_≈0.842. Accordingly, the capacity dimension is *D*_0_≈1.188. The hierarchical correlation pattern of Indian cities is displayed in Figure S4. The correlation dimension is *D*_2_≈1.207, and the goodness of fit is around *R*^2^=0.958. According to equation (20), the Zipf correlation dimension is estimated as *d*_2_≈0.813.

Apparently, for the 300 Indian cities, the Pareto dimension spectrum is not normal because *D*_2_>*D*_0_. However, the Zipf dimension spectrum is acceptable since *d*_2_<*d*_0_. This suggests that the Zipf effect plays a more important role in Indian urban systems than the Pareto effect. In other words, each Indian city tries to become larger and larger in population size. The result of the Zipf effect is that the Zipf dimension will increase and approach 1. If we apply the method to China’s cities in 2000, the results are similar to those of Indian cities given above. This suggests that, for the developed countries such as America, internal complexity of urban system reaches equilibrium with external complexity approximately. However, for the developing countries such as India and China, the internal complexity of cities has an advantage over external complexity (Table S2).
